# Harnessing real-life experiences: the development of guidelines to communicate research findings on Developmental Coordination Disorder/dyspraxia

**DOI:** 10.1186/s40900-024-00611-0

**Published:** 2024-08-08

**Authors:** Catherine Purcell, Annie Dahl, Judith Gentle, Elisabeth Hill, Amanda Kirby, Abby Mason, Victoria McQuillan, Andrea Meek, Sally Payne, Sally Scott-Roberts, Krystal Shaw, Kate Wilmut

**Affiliations:** 1 School of Healthcare Sciences, Cardiff University, Cardiff, UK; 2 Developmental Coordination Disorder Research Advisory Group, Barry, UK; 3 School of Psychology, Faculty of Health and Medical Sciences, University of Surrey, Surrey, UK; 4 Department of Psychology, City University of London, London, UK; 5 Do-IT Solutions Ltd, Cardiff, UK; 6 Developmental Coordination Disorder Research Advisory Group, Leeds, UK; 7 School of Health & Society, University of Salford, Salford, UK; 8 Developmental Coordination Disorder Research Advisory Group, Caerphilly, UK; 9 Dyspraxia Foundation, Hitchin, UK; 10 Faculty of Life Sciences and Education, University of South Wales, Pontypridd, UK; 11 Developmental Coordination Disorder Research Advisory Group, Southampton, UK; 12 Department of Psychology, Health and Professional Development, Centre for Psychological Research, Oxford Brookes University, Oxford, UK; 13 Ty Dewi Sant, Cardiff University, Heath Park, Cardiff, CF14 4XN UK

**Keywords:** DCD-UK committee, DCD Research Advisory Group (DCD-RAG), DCD community, Research summaries

## Abstract

Developmental Coordination Disorder (DCD), also known as dyspraxia, affects 5–15% of school-aged children (Hamilton and Sutton, Am Fam Physician 66:1435, 2002) and significantly impacts a child’s ability to learn motor skills and perform everyday activities efficiently and effectively (Zwicker et al., Eur J Paediatr Neurol 16:573–81, 2012). These motor deficits can have a negative impact on academic performance, vocational choices and leisure pursuits (Zwicker et al., Eur J Paediatr Neurol 16:573–81, 2012) and profoundly impact quality of life (Izadi-Najafabadi et al., Res Dev Disabil 84:75–84, 2019). DCD persists into adulthood (Kirby et al., J Adult Dev 18:107–13, 2011), impacting motor as well as emotional and behavioural status (Tal Saban and Kirby, Curr Dev Disord Rep 5:9–17, 2018). Despite the continued increase in research in the field of DCD, awareness of DCD remains poor (O’Kelly NL., From invisibility to invincibility: Guidelines for supporting families through the diagnosis and journey with developmental coordination disorder, 2012) even though it has higher prevalence rates when compared to, for example, autism spectrum disorder (Yan et al., J Autism Dev Disord :1–7, 2024), which in part may be due to a lack of accessible research findings. A fundamental feature of the research process is disseminating research findings. This should involve community members in design and delivery to ensure the accessibility of research findings.

In 2022 the DCD-UK committee established a DCD Research Advisory Group (DCD-RAG) which met over the course of 12 months to: (1) identify issues of inaccessible research findings; (2) determine the need for a repository for research summaries; (3) co-create guidelines for authors and (4) agree a process for reviewing research summaries to be housed on the Movement Matters website. The new co-produced research repository, author guidelines and process were launched at the DCD-UK conference in Manchester 2023 and subsequently shared on social media and through the DCD research email list. The creation of the DCD-RAG and the process that we undertook together to create a non-academic repository for DCD research summaries are described. It is hoped that this repository will enable the wider public, community members and professionals to be able to readily benefit from accessible research, increasing a deeper and broader understanding of the evidence in the field.

## Abstract (plain language summary)

Developmental Coordination Disorder (DCD) is also known as dyspraxia. DCD can affect a person’s coordination, how they move and how they perform daily activities. It can impact quality of life and social and emotional wellbeing.

Awareness of DCD is poor, so the DCD-UK committee started a Research Advisory Group, called DCD-RAG which includes adults with DCD and parents of children with DCD, to help them make information from DCD researchers more accessible.

The group helped to identify the problems with gaining access to DCD research and they helped to write guidelines for researchers. They helped develop a website called Movement Matters for DCD researchers to file accessible summaries of their work. The group looked at how they could review summaries to ensure good practice.

It is hoped the new website will help make DCD research from both the UK and internationally easier for the public and professionals to find, read and understand.

Public involvement in research can be tokenistic. It is important that researchers allocate time to share findings with the people whose lives are the focus of the research.

This article outlines how co-production with the public can improve the accessibility of research findings. Doing this in a way which is meaningful and accessible should be the minimum that researchers aim for.

## Background

Developmental Coordination Disorder (DCD) or dyspraxia is characterised in childhood by difficulties in motor learning and performing accurate movements that affect activities of daily living and academic achievement [1]. European guidelines were developed in 2019 which outlined current understanding of DCD. These research-informed guidelines, written by academics predominately for other academics and clinicians, provide a thorough overview of DCD along with recommendations that consider five key areas: mechanisms, assessment, intervention, psycho-social and adolescents/adults [2]. These guidelines are important as the prevalence rates for DCD is high, ranging from 2 to 20% of children, with 5% to 6% being the most frequently cited percentage in the literature [1, 3]. DCD also frequently co-occurs with autism spectrum disorder [4] and attention deficit hyperactivity disorder [5]. In many cases DCD persists well into adolescence and adulthood [6–8], with 50% to 70% of children continuing to have motor difficulties [9]. Despite a prevalence rate equivalent to attention deficit hyperactivity disorder and higher than autism spectrum disorder, DCD is less well understood in wider society [10] by doctors [11], teachers [12], employers [13] and the general public. This has led to a call for action to raise awareness of DCD, driven by frustrations from parents about the lack of awareness amongst professionals and the general public [14].

As is the case for much research, the involvement of the community has been limited. However, in 2019 the UK Standards for Public Involvement [15] were launched to improve the quality and consistency of public involvement in research. Public and patient engagement (PPE) is now considered best practice to ensure that research is relevant, accessible and accountable to its end users [16]. Public involvement can be achieved in multiple ways by researchers and most major research funders require and reward meaningful public involvement in bid development and implementation plans, including public co-applicants [17]. Importantly, researchers also typically carry the responsibility for conducting knowledge translation. They should, however, only be the messenger when they have credibility with the target audience, possess the skills and experience needed to transfer research knowledge effectively to the audience and have the time and resources to facilitate access to research [18]. Therefore, whilst community involvement in research is a relatively recent development in the UK, community involvement is now rewarded by funders. Researchers are responsible for ensuring they have the necessary credibility, skills, experience and resources to achieve widespread knowledge translation.

In recognition of this need to evolve, the DCD-UK committee, which was originally founded in 2004 by a group of academics working within the field of DCD research, underwent an overhaul in 2021. Group membership changed and was made up of eight individuals comprising seven academics and a representative from the Dyspraxia Foundation (the UK charity for DCD which ceased trading on the 20th April 2024), all of whom work within the field of DCD / dyspraxia, four members are also clinicians. In 2021 the DCD-UK committee identified the need to co-create its remit with the community and set about recruiting a DCD Research Advisory Group (DCD-RAG). Here we describe the creation of the DCD-RAG and the process that we undertook together to create a non-academic repository for DCD research summaries.

## Framework of research advisory group

### Formation of the DCD-RAG

**Fig. 1:**
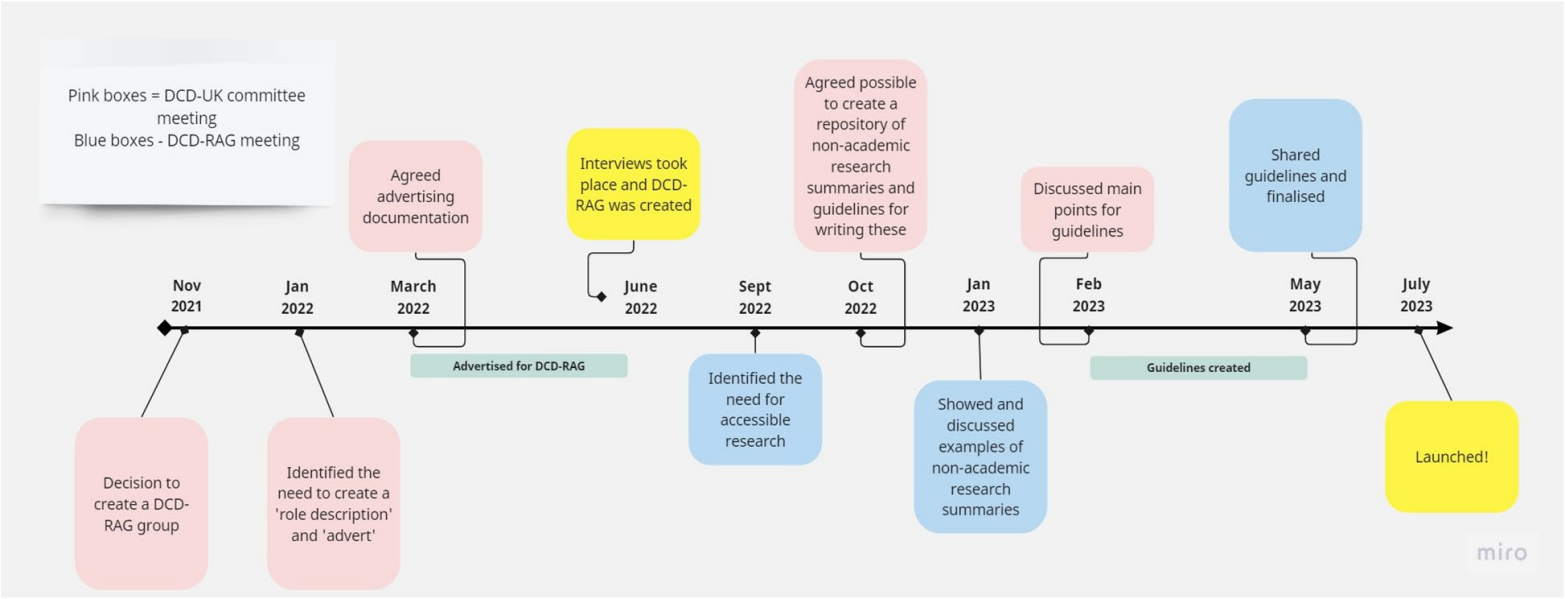
Summary of the timeline

In order to recruit the DCD-RAG we identified the need to create a role description and advert, this included a clear statement outlining that the DCD-RAG would assist, support and advise researchers and act as a ‘critical friend’ on how best we can improve research strategy, projects and dissemination of research findings in the UK. It included a set of responsibilities for the DCD-RAG, a person specification along with the responsibilities of the DCD-UK committee, which included a collective responsibility for sharing decisions made with the wider DCD community. Adverts were placed on social media and distributed to DCD-UK committee members contacts. In line with the payment rates set out by the National Institute for Health and Care Research, applicants were also informed that they would be remunerated for their time. Applications were received between March 2022 and June 2022 and four adults with DCD and two parents of children with DCD were interviewed and agreed to form the DCD-RAG (please see Fig. 1 for a summary of the timeline) in July 2022 for an initial period of two years.

### Initial meeting

The first meeting with the DCD-RAG was held in September 2022 with two academics from the DCD-UK committee. During this meeting we agreed that decisions would be made through open and transparent discussion with a majority vote, if necessary. DCD-RAG members were also asked to consider issues relating to the accessibility of research findings as an initial activity in their role. They highlighted many factors that led to the inaccessibility of research including: the paywall associated with many academic journal articles making them hard to access, the way in which information is presented in academic journals, the fact that many academic journal articles highlight issues associated with DCD but rarely present solutions or strengths and the inaccessible language used in academic journals. This was supported by the feeling that papers focusing on lived experience or co-production were easier to engage with. These issues are not unique to DCD research and have been reported in relation to research of relevance to nurses [19] and autistic adults [20].

### Agreement to create a research repository

Given the challenges expressed by the DCD-RAG members in accessing research, in October 2022 DCD-UK Committee members met with the DCD-RAG for the second time and agreed to create a repository for research summaries on a relaunched and repurposed Movement Matters website, a site originally created by the DCD-UK Committee to house resources. During this meeting we also agreed that a social media presence was needed to promote new research summaries that were added to the research repository and that a set of criteria were needed in order to ensure accessibility of the content and format of research summaries.

### Creation of non-academic summary guidelines

In January 2023 DCD-UK Committee members met with the DCD-RAG having provided example summaries beforehand for discussion (these included two infographics and one ‘lay summary’ from a published paper). During this discussion it became clear that language is important. Language needs to be accessible, avoiding jargon and academic terminology, with attention paid to the framing and tone of how research findings are presented to the wider community. The importance of language has been highlighted in a previous study that emphasised that communication of research requires a process of interpreting or translating often complex research findings into a language, format and context that non-experts can understand [21]. It also became apparent during this meeting that transparency is important: The DCD-RAG felt that included research summaries did not necessarily need to be limited to published research, especially given the length of time the publishing process can take. They felt that unpublished research should include a disclaimer that a peer review process had not yet been undertaken and some mechanism included to ensure that the authors would update the research summary once the findings had been published. The DCD-RAG identified that research summaries should include contact details for the corresponding author, that research summaries should focus on what is important and include recommendations.

During the next DCD-UK Committee in February 2023, we agreed that guidelines were needed for researchers that provided clarity around what was expected of research summaries, for example a statement confirming that ethical approval had been granted and that data collection was complete. It was agreed that the guidelines would also include recommendations regarding the format and language accessibility of the summaries, including length and presentation style including colour contrasts, type face and no longer than 500 words or five minutes long, remembering that some members of the community may use read aloud software. In terms of process, it was agreed that all summaries would need to be checked by a member of the DCD-UK committee and a DCD-RAG member prior to inclusion on the Movement Matters website.

### Drafting and approving guidelines and processes

Following this meeting, the DCD-UK committee wrote a set of draft guidelines for authors, which were shared with the DCD-RAG in March 2023. During the next meeting with the DCD-RAG in May 2023, amendments were made to the guidelines for authors including requesting that authors should update their summaries when the findings had been published and a process for reminding authors was agreed. It was also agreed that we would maintain a spreadsheet indicating when research summaries had been submitted and that this would be updated throughout the review process. We agreed to create email templates and checklists for both members of the DCD-UK committee and DCD-RAG. Finally, we agreed the process: (1) a member of the DCD-UK committee should check the Movement Matters email during the first week of each month for new and amended submissions, submissions should be logged on the spreadsheet and a checklist completed to ensure compliance with the author guidelines; (2) assuming compliance, summaries should then be sent to the DCD-RAG to check accessibility and feedback should be provided to the DCD-UK committee via an online form; (3) a member of the DCD-UK committee should then either post the summary on the Movement Matters website and social media platform or return it to the authors with requested amendments; (4) once amendments have been received these should be checked by the DCD-UK committee and DCD-RAG before being posted on the website and social media platform.

### Finalising and launching the repository

In May 2023 the DCD-RAG discussed any final amendments to the author guidelines and process and agreement was reached to organise the findings under categories on the Movement Matters website. The guidelines and process were then launched at the DCD-UK Conference in Manchester in July 2023 with the DCD-RAG providing video clips for the launch. These explained for example why producing accessible research summaries is important. The repository has since been advertised on social media and through a DCD research email list.

## Summary

Public involvement in research processes can be insufficient, inconsistent, or tokenistic [17, 22]. It is hoped that through this collaborative process the translation of research findings will become more accessible to the wider DCD community. Disseminating research findings facilitate opportunities to explore implications from a local perspective to identify their transferability [23], potentially reducing the gap between research and practice [24]. Community dissemination should therefore involve a two-way dialogue that enables community interpretation to form part of an iterative research process [25]. This process is not without its limitations, for example it is important to acknowledge and address implicit and explicit expectations and ensure that the limited resources of both the DCD-UK Committee and DCD-RAG are carefully managed. It is however hoped that, by introducing a reciprocal relationship to ensure the sharing of research findings in accessible formats, a broader understanding of DCD will be enhanced and it will encourage the community to participate in research studies to facilitate higher quality research and foster greater trust between researchers and the community.

## Reflections from the DCD-RAG in their own words

The DCD-RAG, who co-authored this paper, have provided some reflections about their experiences of being involved in their own words which comprise the remainder of this section.

Advisory groups make sure that researchers listen to the people who have actual experience and who can talk about it in a non-academic way. This is so important for the direction and the dissemination of research. It stops assumptions being made and it allows for people to make a direct impact on research that, if properly done, may make a positive impact on their lives. Sometimes it can be alienating when you are the person who has the condition / life experience that's being researched. It can feel like people are talking about you, at a distance and in a way that isn't particularly relatable to your lived experience. A lot of the public facing literature about dyspraxia is so negative and I would hesitate in sharing most of it because it gives the impression that people with the condition are incapable of doing anything. Therefore, working together makes the research more impactful.

The experience has been really positive. It feels like the whole group are on a very equal footing and there is no "them" (researchers) and "us". It's been an opportunity for us to challenge assumptions around DCD research and to ensure accessibility of research findings. I'm hopeful this good practice will continue and spread to other research groups. Being involved has also helped me to develop confidence to share ideas, which has had a positive impact on my work and personal life. I've also learnt more about dyspraxia from the researchers which has helped me to understand myself better. Being part of a RAG is a step towards delivering research findings more inclusively and in a way that is more reflective of the experiences of people with dyspraxia. Sometimes this is simply through the conversations that we have with academics leading the group and sometimes this is through the work that we produce as a group. That's not just important for the group members but it's also important for the wider dyspraxia community who hopefully will read the research digests. It's also important that the discussions we have with researchers translate into more inclusive opportunities for people living with dyspraxia to access the papers, conferences and research briefs that will impact on our lives.

Living with dyspraxia is really difficult partly because it's so poorly misunderstood and articulated. As someone living with dyspraxia I still haven't found the right way of describing it to my family friends and colleagues without feeling very vulnerable. I think because of this I really enjoy the time in our sessions working alongside very capable, insightful and creative people who also have dyspraxia and the unspoken understanding between us all. We are now in our second year and, as a group, I would like us to become more strategic in how we are approaching the work. There seems to be a consensus in the group that our outputs should be more consistent to reflect our inclusive ethos so that the work we produce is high quality and accessible. At our most recent meeting we talked about setting some goals for the year ahead and I think this will really help to propel the enthusiasm of all our group members and make a difference to the dyspraxia community. I'm hopeful that researchers will think about the accessibility and inclusivity of their research—right from the start to the very end. Opportunities for people with lived experience of DCD to influence and engage with researchers should be standard across all projects. There needs to be a realisation from researchers and universities that academic journals are not always the best forms of impact. Accessible versions of findings should be standard, an infographic, a video or an easy read document I can stick to my fridge are much more likely to make me engaged with research and therefore have more of an impact on my knowledge and awareness of DCD. Long term., I hope that research will not only be influenced by people with lived experience but that they will lead it, decide methodologies and distribute funding to ensure it has people with dyspraxia at the heart of it and it makes a difference. Short term, I hope researchers engage with the Movement Matters website to make their work more accessible.

## Recommendations

Given our interaction with the DCD-RAG we would fully support the creation of a non-academic repository for any research involving human participants, especially those involving clinical populations. Ensuring that findings are accessible to community members and other non-academic interested parties should be at the heart of research. Furthermore, we firmly believe that consideration must be given to the format and content of a non-academic summary and that many of the lay summaries published alongside journal articles do not meet the minimum guidelines our DCD-RAG laid out. In short, a non-academic summary should:

1. Be written/spoken in accessible language
2. Be framed in positive, non-judgemental language
3. Be standalone in content (i.e. not rely on specialised knowledge)
4. Be accessible to an e-reader or include closed/open captions
5. Be linked to the full article/researcher contact details
6. Include a clear statement regarding whether the content is published (peer reviewed) or not
7. Ideally be approved prior to publication by a stakeholder non-academic group

An outline of the full guidelines created in this process can be found at the Movement Matters Website—https://movementmattersuk.org/.

## Conclusions

As academics working within busy research institutions, we realise that time is of a premium. However, the research that we do matters to so many different individuals with varying levels of time, resources and academic knowledge. If we truly want our research to impact the lives of the people on which it focuses it is imperative that we allocate time to disseminate our findings back to those who took part, to the wider community and to society in general. Doing this in a way which is meaningful and accessible to those individuals should be the minimum we aim for. We encourage groups or individuals to make use of the guidelines we have created for DCD in conjunction with DCD-RAG.

## Data Availability

No datasets were generated or analysed during the current study.
